# Differential Iterative Joint Estimation Approach for Indoor Target Localization

**DOI:** 10.3390/s26113442

**Published:** 2026-05-29

**Authors:** Zhigang Su, Jingyuan Xu, Jingtang Hao, Bing Han

**Affiliations:** Sino-European Institute of Aviation Engineering, Civil Aviation University of China, Tianjin 300300, China; 191543740@cauc.edu.cn (J.X.); jthao_siae@126.com (J.H.); b-han@cauc.edu.cn (B.H.)

**Keywords:** indoor localization, received signal strength indicator, Differential Iterative Joint Estimation, path-loss exponent, eigenvalue method

## Abstract

To address the sharp degradation in positioning accuracy and the lack of robustness of received signal strength indication (RSSI)-based indoor localization methods when both the reference RSSI and path-loss exponent are mismatched, a Differential Iterative Joint Estimation (DIJE) localization method is proposed in this paper. The proposed method first employs a differential model to eliminate the uncertainty caused by reference RSSI, transforming the maximum likelihood estimation (MLE) problem into a matrix eigenvalue problem to enable fast and high-accuracy target position estimation. Additionally, an alternating iterative optimization framework for target position and path-loss exponent is constructed to achieve adaptive joint estimation of model parameters and target coordinates, effectively suppressing localization performance degradation induced by parameter mismatch. In this paper, the Cramér–Rao Lower Bound (CRLB) under the dual-parameter uncertainty scenario is derived as a theoretical performance benchmark, and both simulation experiments and public real-world datasets are used to validate the method’s performance. The results demonstrate that the DIJE method can approach the theoretical limit under varying noise levels, access point (AP) densities, and complex indoor environments. Compared with classical algorithms such as RSDPE, MLE-TLLS, SOCP3, and LCJE, the DIJE method exhibits significant advantages in localization accuracy, robustness, and adaptability to initial parameters, and can meet the engineering requirements of high-accuracy and low-latency real-time indoor localization.

## 1. Introduction

With the rapid development of the Internet of Things (IoT) and intelligent space technologies, indoor localization has emerged as a critical enabling technology for scenarios including smart retail, medical monitoring, industrial operation and maintenance, and public safety [1,2]. High-accuracy and high-robustness location services are of great significance for enhancing operational efficiency, strengthening safety assurance, and optimizing user experience [3,4]. In recent years, indoor localization technologies have developed along multiple technical routes, including radio-frequency-based methods using Wi-Fi, BLE, UWB, RFID, and millimeter-wave signals, as well as approaches based on inertial sensors, vision, ultrasound, and multi-source fusion [5]. Among these technologies, Wi-Fi localization has become an attractive solution for large-scale indoor positioning systems because of its widespread device availability, low deployment cost, and no requirement for additional hardware modification. Device-free localization (DFL) based on Wi-Fi signal perturbations does not require the target to carry any positioning device; instead, it estimates the position by exploiting the perturbations caused by a human or target to the wireless channel. Such methods typically employ channel state information (CSI) as the signal feature and can incorporate online learning, domain adaptation, or multiple kernel learning to enhance robustness in complex environments [6,7]. In contrast, the logarithmic path-loss localization method based on received signal strength indication (RSSI) can directly extract signal features from Wi-Fi broadcast frames. Owing to its simple implementation, low computational cost, and clear physical interpretation, it has been widely used in low-cost indoor localization systems.

Indoor localization methods based on RSSI can generally be divided into two categories: those based on the propagation model [8] and those based on fingerprint databases [9]. Propagation-ranging-model-based methods estimate the distance between the target and each access point (AP) by converting the received RSSI measurements according to the log-distance path-loss model, and subsequently they determine the target location by exploiting the geometric distance relationships between the target and multiple APs. These methods are favored due to their clear physical meaning, low computational complexity, and portability in measurement system setup. As a result, they have long attracted the attention of scholars. However, such methods heavily depend on model parameters and tend to have lower localization accuracy. On the other hand, fingerprint-based methods require the pre-construction of a fingerprint database for the localization area offline, and during the online phase, the collected RSSI fingerprints are matched with the samples in the database using methods such as KNN, support vector machines, random forests, neural networks, or deep learning techniques to achieve localization [10]. In recent years, machine learning and deep learning methods have been applied to RSSI fingerprint-based localization due to their ability to learn complex nonlinear signal features. These methods generally perform well in scenarios where sufficient training data is available. However, the major limitation of fingerprint-based methods is the need for a large amount of offline calibration data when constructing the fingerprint database. The collection and updating of such data introduce significant human, material, and time costs, limiting the convenience of their application.

Considering the feasibility of deploying the localization system in large indoor areas, this study focuses on methods based on the propagation model and explores ways to improve the positioning accuracy of traditional RSSI-based localization methods in practical scenarios.

Traditional RSSI-based localization methods primarily adopt approaches such as maximum likelihood estimation (MLE) [11,12,13,14,15], and their localization accuracy can approach the Cramér–Rao Lower Bound (CRLB) under ideal conditions. However, such methods are highly dependent on the accuracy of the RSSI at the reference distance and the path-loss exponent. In practical indoor environments, these parameters vary dynamically over time and space due to wall obstruction, human movement, multipath propagation, and device heterogeneity. Consequently, parameters obtained via offline calibration or empirical presetting are difficult to match the actual propagation environment. Parameter mismatch introduces systematic ranging errors, which directly result in a sharp degradation in localization accuracy and a significant deterioration in robustness, thus becoming a core challenge that restricts the engineering implementation of RSSI-based localization.

In addressing the joint localization problem under parameter uncertainty, researchers have proposed improved methods such as second-order cone programming (SOCP) [16], semidefinite programming (SDP) [17], differential RSSI [18], and weighted least squares [19]. The SOCP method iteratively solves for both the target location and the path-loss exponent based on second-order cone optimization. This approach addresses the target localization problem when the initial path-loss exponent value in the log-distance path-loss model deviates from the true value, but it still requires prior knowledge of the reference RSSI in the model. The SDP method transforms the non-convex joint estimation problem into a convex semidefinite programming problem through semidefinite relaxation, simultaneously estimating the target location and the path-loss exponent. The advantage of the SDP method lies in its independence from initial values and its ability to converge to the global optimum. However, its drawbacks include high dimensionality due to joint estimation, high computational complexity, poor real-time performance, and the need for prior knowledge of the reference RSSI in the model. The differential RSSI method eliminates common errors between APs by taking differences between pairwise RSSI measurements and then constructs a set of linear direction constraints using the obtained distance ratio constraints, ultimately localizing the target via linear least squares. This method does not require prior knowledge of the reference RSSI and imposes low precision requirements on the initial path-loss exponent. However, the use of distance ratio constraints results in the loss of absolute distance information, leading to lower theoretical localization accuracy. The weighted least squares method applies weighted least squares processing to the linearized equation system to jointly estimate the target location and the path-loss exponent, yielding a low-complexity, non-iterative closed-form solution. Nevertheless, this method still requires prior knowledge of the reference RSSI in the model, and the model error introduced by linearization makes it less robust to noise.

In summary, existing joint localization methods for parameter-uncertain scenarios are either sensitive to initial parameter settings, computationally expensive, or insufficiently stable under strong noise and complex environmental conditions, making it difficult to simultaneously achieve high accuracy, low latency, and strong robustness. To address the more challenging problem of high-accuracy target localization under the dual mismatch of reference RSSI and path-loss exponent, this paper proposes a Differential Iterative Joint Estimation (DIJE) localization method. The DIJE method employs a differential model to eliminate the uncertainty introduced by the reference RSSI, and incorporates an eigenvalue decomposition mechanism to enable fast target position estimation under a mild linearization approximation. This avoids the high computational complexity of SDP/SOCP-based relaxation methods and the large approximation errors associated with differential RSSI and weighted least squares methods, thereby achieving a balance between localization accuracy and computational efficiency. Furthermore, by alternately updating the target position and the path-loss exponent, DIJE realizes adaptive joint estimation of the model parameter and target location, significantly suppressing the performance degradation caused by model mismatch. The primary contributions of this work are summarized as follows:

- A differential eigenvalue-based position estimation method is developed. By leveraging a differential model to eliminate the uncertainty of the reference RSSI, and integrating generalized least squares with eigenvalue decomposition, fast and high-precision localization is achieved.
- An iterative joint estimation framework for the target position and path-loss exponent is constructed. Through alternating optimization, dynamic adaptation of model parameters is realized, which significantly improves the robustness of the localization method under parameter mismatch scenarios.
- The CRLB for the dual-parameter uncertainty scenario is derived, and the performance of the proposed DIJE method is validated via both simulations and real-world datasets. The results demonstrate that the DIJE method can approach the theoretical optimal performance under diverse noise levels, AP densities, and complex indoor environments.

The remainder of this paper is organized as follows. Section 2 establishes the RSSI localization model and the differential model, and analyzes the impact of parameter mismatch on localization performance. The target position solution method based on differential eigenvalues is presented in Section 3. Section 4 introduces the generalized least squares estimation of the path-loss exponent. Section 5 elaborates on the iterative joint estimation procedure of the DIJE method in detail. The theoretical performance lower bound is derived in Section 6. Section 7 and Section 8 validate the performance of the DIJE method through simulations and public datasets, respectively. Finally, the conclusions and future research directions are summarized in Section 9. Table 1 lists the key notations and their definitions used throughout the paper.

## 2. Model Derivation and Methodology

### 2.1. RSSI-Based Localization Model

**Figure 1:**
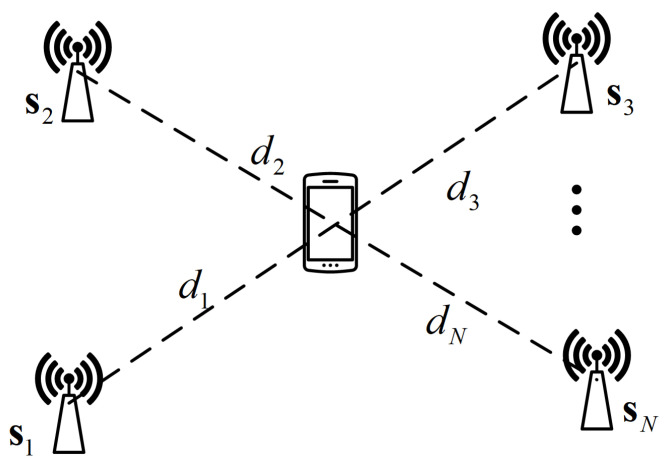
Topology of RSSI-based target localization in a WSN.

Consider a Wireless Sensor Network (WSN) composed of *N* APs with known coordinates, whose topology is illustrated in Figure 1. Let $s_{1},s_{2},\cdots ,s_{N}$ denote the two-dimensional position vectors of the *N* APs, where the position vector of the *n*-th AP is explicitly given by $s_{n}={\left[x_{n},y_{n}\right]}^{T}$. The position vector of the target to be localized is defined as $u={\left[x,y\right]}^{T}$. The Euclidean distance $d_{n}$ between the target and the *n*-th AP is expressed as

(1) $$d_{n}=∥u-s_{n}∥.$$

In the WSN, each AP receives the Wi-Fi signal transmitted by the target, and estimates the distance $d_{n}$ based on the RSSI measurements. The target position is then solved via cooperative computation among multiple APs.

In indoor localization scenarios, Wi-Fi signals transmitted by the target are typically subject to multiple propagation impairments, including obstruction, reflection, and scattering caused by indoor furniture, walls, human occupants, and other obstacles. Consequently, the actual signal attenuation rate far exceeds the prediction of the free-space propagation model. To address the challenge of accurately modeling signal propagation loss in complex indoor environments, the logarithmic path-loss model is proposed based on the free-space model, by introducing the path-loss exponent and the log-normal shadowing term [20]. In RSSI-based localization, since path-loss is directly correlated with RSSI measurements, the logarithmic path-loss model can be transformed into a functional relationship between RSSI and distance,

(2) $$r_{n}=r_{0}-10\eta \mathrm{lg}\frac{d_{n}}{d_{0}}+v_{n},$$

where $r_{n}$ denotes the received RSSI of the target signal at the *n*-th AP, and $r_{0}$ represents the average RSSI of the signal transmitted by the target at the reference distance $d_{0}$ from the AP, which is hereafter referred to as the reference RSSI. The parameter η is defined as the path-loss exponent, which quantifies the rate of signal strength attenuation with increasing propagation distance. Meanwhile, $v_{n}$ stands for the shadow fading random variable, following a zero-mean Gaussian distribution. For typical indoor localization scenarios, the reference distance is commonly set to $d_{0}$ = 1 m. Consequently, Equation (2) can be simplified as

(3) $$r_{n}=r_{0}-10\eta \mathrm{lg}d_{n}+v_{n}.$$

In a WSN, the shadow fading terms $v_{n}$ corresponding to different APs are assumed to be independent and identically distributed (i.i.d.). In practical engineering environments, for scenarios that deviate from the strict i.i.d. assumption, various calibration techniques can be employed to refine the signal model. These include regional partitioning modeling, correlation calibration, and dynamic access point selection, thereby ensuring the shadow fading process adheres to the i.i.d. property. Without loss of generality, let the standard deviation of the shadow fading term $v_{n}$ be σ; then, $v_{n}$ is a zero-mean Gaussian random variable with variance $\sigma^{2}$. To simplify the localization problem, RSSI-based indoor localization methods commonly assume that all APs share the same reference RSSI and a single path-loss exponent [21]. This assumption remains valid for typical AP deployments and within local regions, accurately reflecting the target position and signal attenuation characteristics.

As can be observed from Equation (3), in RSSI-based localization for WSNs, the path-loss exponent η, reference RSSI $r_{0}$, and shadow fading term $v_{n}$ constitute the three core parameters of the logarithmic path-loss model. Errors in these parameters are directly propagated to distance estimation, thereby degrading the final target localization accuracy. Specifically, the path-loss exponent and reference RSSI introduce systematic errors, while the shadow fading term introduces random errors.

In conventional range-based WSN localization, the path-loss exponent and reference RSSI are required to be known a priori. The RSSI measurements are first converted into distance estimates between the target and each AP via the logarithmic path-loss model, and the target coordinates are then computed using geometric algorithms based on the obtained distance information. When the path-loss exponent and reference RSSI are subject to errors, calibration or parameter estimation techniques must be employed to mitigate the impact of these parameter errors on system accuracy and enhance the target localization performance.

### 2.2. Eliminating the Effect of $r_{0}$ via the Differential Method

In the logarithmic path-loss model, $r_{0}$ denotes the average received signal strength of the target signal at the reference distance $d_{0}$ from the AP. This parameter comprehensively characterizes the combined effects of transmit power, antenna gain, propagation loss at the reference distance, and other related factors. In WSN localization, the accuracy of $r_{0}$ directly determines the baseline precision of distance estimation, and its consistency and stability are critical to the overall performance of the localization system. However, due to the influence of hardware imperfections, environmental variations, and measurement conditions, the $r_{0}$ values across different APs cannot be simply assumed to be identical. In engineering practice, strategies such as hardware standardization, precise calibration, and algorithmic compensation can be implemented to achieve the effect of treating the $r_{0}$ values of different APs as identical.

For the WSN localization problem investigated in this paper, we assume that the $r_{0}$ values of all APs are identical. For any individual AP whose reference RSSI deviates from $r_{0}$, the corresponding offset can be absorbed into the shadow fading term $v_{n}$, which alters the mean value of $v_{n}$. Therefore, to eliminate the influence of $r_{0}$, a differential method is employed across different APs. Taking the RSSI received at the first AP as the reference, the RSSI measurements at the remaining APs are differenced with it in accordance with Equation (3):

(4) $$r_{n}-r_{1}=10\eta \mathrm{lg}d_{1}-10\eta \mathrm{lg}d_{n}+\varepsilon_{n},$$

where $\varepsilon_{n}=v_{n}-v_{1}$ denotes the noise component after the differencing operation. Using the noise components from the N−1 differential equations, the noise vectors are constructed as $\varepsilon ={\left[\varepsilon_{2},\varepsilon_{3},\cdots ,\varepsilon_{N}\right]}^{T}$, whose covariance matrix is given by

(5) $$C_{\varepsilon }=\sigma^{2}\left(I_{N-1}+1_{N-1}1_{N-1}^{T}\right),$$

where $I_{N-1}$ is the (N−1)-dimensional identity matrix, and $1_{N-1}$ is the (N−1)-dimensional all-one column vector. As can be observed from Equation (5), the noise components are no longer mutually independent after the differencing operation. Define the residual of Equation (4) as

(6) $$g_{n}=\left(r_{n}-r_{1}\right)-\left(10\eta \mathrm{lg}d_{1}-10\eta \mathrm{lg}d_{n}\right).$$

The residual vector is then defined as $g={\left[g_{2},g_{3},\cdots ,g_{N}\right]}^{T}$, based on which the cost function for the generalized least squares (GLS) method is constructed as

(7) $$J_{1}\left(u\right)=g^{T}C_{\varepsilon }^{-1}g.$$

This cost function weights the residuals using the inverse of the noise covariance matrix $C_{\varepsilon }$, thereby eliminating the impact of noise correlation. The estimate of the target position u is obtained by minimizing the cost function $J_{1}\left(u\right)$.

### 2.3. Effect of Path-Loss Exponent Uncertainty on Localization Performance

The path-loss exponent η characterizes the rate at which signal strength attenuates with propagation distance. When η deviates from its true value, it introduces a systematic and globally adverse impact on the localization accuracy of WSNs. The bias in η propagates through the logarithmic path-loss model, manifesting as systematic errors in distance estimation. Critically, this error grows significantly as the distance between the target and the AP increases, ultimately leading to biased or distorted target position estimates. As a systematic error, this degradation cannot be eliminated by averaging multiple measurements, making it one of the core error sources in long-range range-based localization.

Theoretical analysis demonstrates that the localization error of a WSN is positively correlated with the relative error of the path-loss exponent. Through strategies such as precise calibration and deployment optimization, the relative error of the path-loss exponent can be controlled within 5–10%, thereby ensuring that the localization accuracy of the WSN meets application requirements. To verify this conclusion, in this paper, six APs are deployed in a 30 m × 30 m localization area, where one AP is placed at the center of the area, and the remaining five APs are uniformly distributed on a circle centered at the area center with a radius of 12 m. The localization area is divided into 1 m × 1 m grids, and the target is sequentially placed at the center of each grid to conduct localization experiments.

In the experiments, the true path-loss exponent is set to η = 4, the reference RSSI $r_{0}$ is set to −40 dBm, and the standard deviation σ of the shadow fading ranges from 1 dB to 6 dB. For a given estimated path-loss exponent $\eta_{0}$, the ETL method [22] is employed to perform 100 Monte Carlo localization experiments for the target at each grid center. A total of 900 grids are tested, resulting in 90,000 experiments in total. Based on the above localization results, the total mean square error (TMSE) of target localization under a given $\eta_{0}$ is calculated. Let the position mean square error at the i-th test location be denoted as ${\mathrm{MSE}}_{p}\left(i\right)$. Then, ${\mathrm{TMSE}}_{p}$ is defined as

(8) $${\mathrm{TMSE}}_{p}=\frac{1}{M_{t}}\sum_{i=1}^{M_{t}}{\mathrm{MSE}}_{p}\left(i\right).$$

**Figure 2:**
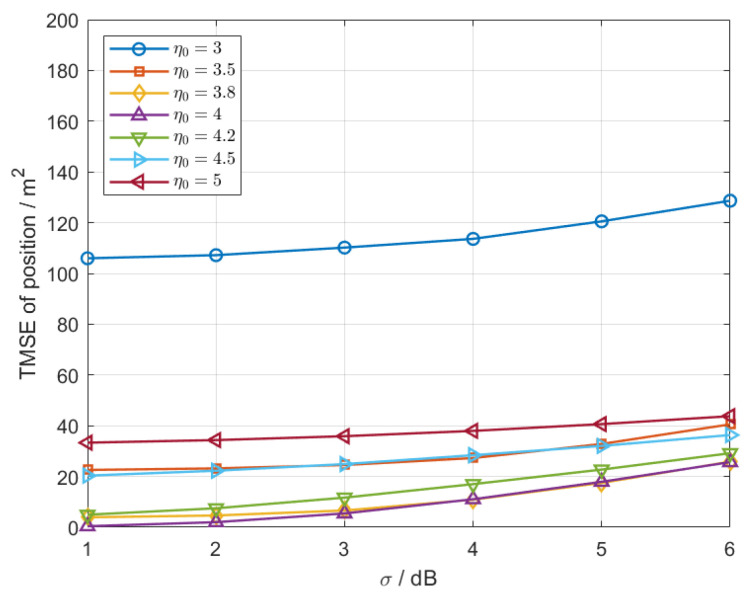
The impact of inaccurate path-loss exponent on localization accuracy with σ.

Let $M_{t}=900$ denote the total number of test locations, which is utilized to evaluate the localization performance of the WSN across the entire coverage area. For different values of the estimated path-loss exponent $\eta_{0}$, the variation curves of ${\mathrm{TMSE}}_{p}$ versus the shadow fading standard deviation σ are illustrated in Figure 2.

As illustrated in Figure 2, when the estimated path-loss exponent $\eta_{0}$ aligns exactly with the true value η = 4, the ${\mathrm{TMSE}}_{p}$ remains at its minimum across the entire range of shadow fading standard deviations σ, signifying optimal localization accuracy. When the relative deviation of $\eta_{0}$ is held within 5% (i.e., $\eta_{0}$ = 3.8 or 4.2), the ${\mathrm{TMSE}}_{p}$ increases moderately from the optimal case, yet the overall increment remains constrained within 5 m^2^. This indicates that moderate biases in $\eta_{0}$ exert limited impact on localization precision, yet are still capable of meeting the accuracy requirements of most indoor application scenarios.

As the deviation of $\eta_{0}$ increases further (e.g., $\eta_{0}$ = 3, 3.5, 4.5, or 5), the ${\mathrm{TMSE}}_{p}$ rises significantly, and the curve profile tends to flatten. This observation reveals that the impact of shadow fading σ is diminished. Consequently, when the path-loss exponent bias is large, the bias itself becomes the dominant factor restricting WSN localization accuracy, which cannot be effectively mitigated by simply increasing the number of repeated experiments.

In summary, the estimation bias of the path-loss exponent directly determines the localization accuracy level of the WSN: a small bias leads to controllable performance degradation, whereas a large bias causes severe deterioration, and such effects are essentially irreversible. Therefore, in practical deployment, the relative error of the path-loss exponent must be strictly controlled at a low level through precise calibration techniques, so as to ensure the overall performance of the localization system.

When a WSN is employed for indoor moving target localization, the relative positions between the target and the APs vary continuously. This induces changes in the signal propagation paths, thereby giving rise to the time-varying characteristic of the path-loss exponent. Any deviation between the preset path-loss exponent and its actual value will directly degrade the localization accuracy of the WSN for moving targets. Therefore, to enhance the localization performance for indoor moving targets, real-time estimation of the path-loss exponent is required.

Theoretically, the propagation path between the target and each AP may differ, and the corresponding path-loss exponents may therefore be different. This implies that multiple unknown path-loss exponents would need to be introduced into the WSN localization model, which would significantly increase the dimensionality of the localization problem and could even render the problem unsolvable. If a single path-loss exponent is adopted, the bias of the log-distance model may increase when the target is located in an environment with severe multipath propagation and dense obstacles, such as metallic obstructions, especially in the presence of numerous non-line-of-sight (NLOS) paths. This may further degrade the target localization performance. However, in practical localization, APs with relatively high RSSI values are usually selected to participate in positioning. Since APs with higher RSSI values are generally closer to the target, the selected APs tend to be concentrated within a relatively small region around the target. Within this local region, the differences among the path-loss exponents of different propagation paths are relatively small. Therefore, the path-loss exponent in the WSN localization model can be treated as a unified parameter, and only this single path-loss exponent needs to be estimated in real time.

### 2.4. Problem Description

As previously discussed, in WSN localization, the reference RSSI $r_{0}$, path-loss exponent η, and shadow fading term $v_{n}$ are the core parameters governing the system’s localization performance. In particular, when η and $r_{0}$ deviate from their true values, the resulting impact on localization accuracy cannot be eliminated by averaging repeated measurements. Instead, calibration or parameter estimation techniques are required to mitigate the effect of these biases on positioning accuracy.

In the WSN localization problem investigated in this paper, we assume that the $r_{0}$ values of all APs involved in localization are identical. Therefore, the influence of $r_{0}$ can be eliminated via the differential method. In practical localization environments, hardware heterogeneity, antenna-gain differences, and local environmental obstructions may cause the reference RSSI of individual APs involved in localization to deviate from $r_{0}$. As described in Section 2.2, this deviation can be incorporated into the shadow fading term and regarded as a change in the mean of the shadow fading $v_{n}$, thereby affecting the mean of the noise component in Equation (4). As indicated by the GLS cost function $J_{1}\left(u\right)$ in Equation (7), the target position estimate depends on the noise covariance matrix $C_{\varepsilon }$, rather than on the noise mean. Therefore, the mean shift introduced into the shadow fading and noise terms by the deviation of $r_{0}$ has no effect on the target position estimation.

To obtain an accurate target position estimate by minimizing $J_{1}\left(u\right)$, the path-loss exponent in Equation (6) must be set to its true value; otherwise, the resulting position estimate will exhibit a systematic bias. In practical scenarios, however, the path-loss exponent adopted in the WSN localization model is typically a preset value, which inevitably deviates from the actual one. Consequently, appropriate methods are required to eliminate this bias. As derived from Equation (3), the bias in η introduces a multiplicative error. Unlike the additive error induced by the $r_{0}$ bias, this error cannot be absorbed into the shadow fading term, thus necessitating a new correction method.

Although the target position estimate $\hat{u}$ obtained under a preset η contains residual error, it can be treated as a known quantity and substituted into Equation (3) in place of u, while η is treated as the unknown parameter to be estimated. Subsequently, with the estimated path-loss exponent $\hat{\eta }$ taken as known, a more accurate target position estimate $\hat{u}$ can be obtained by minimizing $J_{1}\left(u\right)$. By iteratively repeating this alternating process, progressively refined estimates of $\hat{\eta }$ and $\hat{u}$ can be obtained. Therefore, when both the reference RSSI $r_{0}$ and path-loss exponent η are unknown or inaccurately known, the differential method can be employed to eliminate the impact of $r_{0}$, and an iterative alternating optimization scheme can be adopted to jointly refine the target position and path-loss exponent estimates, thereby achieving high-accuracy target localization in the WSN.

## 3. Estimation of the Target Position

Under a preset path-loss exponent η, the WSN estimates the target position based on the received RSSI measurements. As described in Section 2.2, the differential method is employed to eliminate the influence of $r_{0}$, resulting in the signal model in Equation (4). Let $p_{n}=\exp\left(k\cdot r_{n}\right)$ and k=(ln10)/5η. Substituting these into Equation (4) and rearranging it into a natural logarithmic form yields

(9) $$\ln p_{n}-\ln p_{1}=\ln d_{1}^{2}-\ln d_{n}^{2}+k\varepsilon_{n}.$$

Taking $d_{n}^{2}$ as the variable, a first-order Taylor expansion of $\ln d_{n}^{2}$ in Equation (9) at ${\hat{d}}_{n}^{2}$ is performed, which yields

(10) $$\ln d_{n}^{2}=\ln{\hat{d}}_{n}^{2}+\frac{1}{{\hat{d}}_{n}^{2}}\left(d_{n}^{2}-{\hat{d}}_{n}^{2}\right)+o\left(d_{n}^{2}-{\hat{d}}_{n}^{2}\right),$$

where ${\hat{d}}_{n}$ denotes a coarse estimate of $d_{n}$, and $o(d_{n}^{2}-{\hat{d}}_{n}^{2})$ represents the higher-order term, which is neglected in the first-order approximation. The estimate ${\hat{d}}_{n}$ is usually obtained from Equation (3):

(11) $${\hat{d}}_{n}=10^{\left(r_{0}-r_{n}\right)/10\eta \;\;}=\sqrt{p_{0}/p_{n}\;},$$

where $p_{0}=\exp\left(k\cdot r_{0}\right)$. In the absence of shadow fading $v_{n}$, the coarse estimate ${\hat{d}}_{n}$ is theoretically equal to the true distance $d_{n}$. However, when the effect of shadow fading $v_{n}$ is neglected, a bias is introduced between ${\hat{d}}_{n}$ and $d_{n}$, and the magnitude of this bias is positively related to the approximation error caused by shadow fading. When the shadow fading is severe, ${\hat{d}}_{n}$ may deviate from the true value, in which case the first-order Taylor approximation in Equation (10) may introduce an approximation error. This error may affect the target localization accuracy and may lead to a deterioration in the localization performance of the system. Therefore, due to the use of the first-order Taylor approximation in Equation (10), the proposed method is more suitable for environments with relatively weak shadow fading $v_{n}$. Substituting Equations (10) and (11) into Equation (9) and rearranging the terms yields

(12) $$d_{n}^{2}p_{n}-d_{1}^{2}p_{1}=kp_{0}\varepsilon_{n}.$$

Obviously, Equation (12) is an approximate form of the differential model in Equation (4) after Taylor expansion. The subsequent target position estimation is performed based on the approximate model in Equation (12); therefore, the first-order Taylor approximation affects the accuracy of the target position estimate. Nevertheless, as the estimation of the path-loss exponent is refined, a more accurate target position estimate can be obtained from the model in Equation (12). During the iterative process, however, the intermediate target position estimates cannot update the approximate model in Equation (12). Therefore, the influence of the first-order Taylor approximation cannot be reduced through the iterative procedure. Define $h_{n}=d_{n}^{2}p_{n}-d_{1}^{2}p_{1}$. Substituting Equation (1) into $h_{n}$ yields

(13) $$h_{n}=a_{n}u^{T}u-2b_{n}^{T}u+c_{n},$$

where $a_{n}=p_{n}-p_{1}$, $b_{n}=p_{n}s_{n}-p_{1}s_{1}$, and $c_{n}=p_{n}s_{n}^{T}s_{n}-p_{1}s_{1}^{T}s_{1}$. Using the N−1 components $h_{n}$, the vector $h={\left[h_{2},h_{3},\cdots ,h_{N}\right]}^{T}$ is constructed, and its covariance matrix is given by

(14) $$C_{h}=k^{2}p_{0}^{2}C_{\varepsilon }.$$

Based on Equation (12), the GLS cost function for target position estimation is expressed as

(15) $$J_{2}\left(u\right)=h^{T}C_{h}^{-1}h=\frac{1}{k^{2}p_{0}^{2}}h^{T}C_{\varepsilon }^{-1}h.$$

According to the Sherman-Morrison formula, the inverse of $C_{\varepsilon }$ can be expressed as

(16) $$C_{\varepsilon }^{-1}=\frac{1}{\sigma^{2}}\left(I_{N-1}-\frac{1}{N}1_{N-1}1_{N-1}^{T}\right),$$

Substituting Equation (16) into Equation (15) and rearranging the terms yields

(17) $$J_{2}\left(u\right)=\frac{1}{k^{2}p_{0}^{2}\sigma^{2}}\left[\sum_{n=2}^{N}h_{n}^{2}-\frac{1}{N}{\left(\sum_{n=2}^{N}h_{n}\right)}^{2}\right].$$

By minimizing the GLS cost function $J_{2}\left(u\right)$, the following equation is obtained:

(18) $$k_{1}u^{T}\mathrm{uu}+k_{2}u^{T}u+k_{3}\mathrm{uu}+k_{4}u+k_{5}=0,$$

where the corresponding coefficients are defined as

(19) $$k_{1}=\sum_{n=2}^{N}a_{n}^{2}-\frac{1}{N}\sum_{n=2}^{N}a_{n}\sum_{n=2}^{N}a_{n},$$

(20) $$k_{2}=-\sum_{n=2}^{N}a_{n}b_{n}+\frac{1}{N}\sum_{n=2}^{N}a_{n}\sum_{n=2}^{N}b_{n},$$

(21) $$k_{3}=-2\sum_{n=2}^{N}a_{n}b_{n}^{T}+\frac{2}{N}\sum_{n=2}^{N}a_{n}\sum_{n=2}^{N}b_{n}^{T}=2k_{2}^{T},$$

(22) $$k_{4}=\sum_{n=2}^{N}(2b_{n}b_{n}^{T}+a_{n}c_{n}I_{2})-\frac{1}{N}\left(2\sum_{n=2}^{N}b_{n}\sum_{n=2}^{N}b_{n}^{T}+I_{2}\sum_{n=2}^{N}a_{n}\sum_{n=2}^{N}c_{n}\right),$$

(23) $$k_{5}=-\sum_{n=2}^{N}c_{n}b_{n}+\frac{1}{N}\sum_{n=2}^{N}c_{n}\sum_{n=2}^{N}b_{n}.$$

As can be observed from Equation (21), $k_{3}=2k_{2}^{T}$, and both are determined by the AP coordinates and the corresponding RSSI values. Therefore, the coordinate system can be translated such that $k_{2}$ and $k_{3}$ become zero vectors, thereby simplifying Equation (18). Assume that the origin of the new coordinate system is located at $s_{0}$ in the original coordinate system. Then, the position vectors of the target and the APs in the new coordinate system are updated as

(24) $$\left\{\begin{array}{c} v=u-s_{0}\\ s_{n}^{\prime }=s_{n}-s_{0} \end{array}\right.$$

After the coordinate translation, Equation (18) is rewritten in the new coordinate system as

(25) $$k_{1}v^{T}\mathrm{vv}+k_{2}^{\prime }v^{T}v+k_{3}^{\prime }\mathrm{vv}+k_{4}^{\prime }v+k_{5}^{\prime }=0,$$

where $k_{2}^{\prime }$, $k_{3}^{\prime }$, $k_{4}^{\prime }$, and $k_{5}^{\prime }$ are obtained by substituting the AP position vectors in the new coordinate system into Equations (20)–(23). Substituting Equation (24) into Equation (20) and rearranging the terms yields

(26) $$k_{2}^{\prime }=k_{2}+k_{1}s_{0}.$$

Therefore, by choosing

(27) $$s_{0}=-\frac{1}{k_{1}}k_{2}.$$

$k_{2}^{\prime }$ and $k_{3}^{\prime }$ become zero vectors, which simplifies Equation (25) to

(28) $$k_{1}v^{T}\mathrm{vv}+k_{4}^{\prime }v+k_{5}^{\prime }=0,$$

which can be rearranged as

(29) $$v^{T}\mathrm{vv}=-\frac{k_{4}^{\prime }}{k_{1}}v-\frac{k_{5}^{\prime }}{k_{1}}.$$

Taking the Hadamard product of Equation (29) with v yields:

(30) $$v^{T}\mathrm{vv}⊙v=-\frac{k_{4}^{\prime }}{k_{1}}v⊙v-\mathrm{diag}\left(\frac{k_{5}^{\prime }}{k_{1}}\right)v,$$

where ⊙ denotes the Hadamard product, i.e., the element-wise multiplication of two matrices, and diag(·) is the operator that transforms a column vector into a diagonal matrix. From the relationship between the Hadamard product and the vector inner product, we obtain the following:

(31) $$v^{T}v=1_{2}^{T}\left(v⊙v\right).$$

Equations (29)–(31) describe the linear relationships among $v^{T}v$, v⊙v, v, and the constant term. Therefore, the quadratic term v⊙v, the linear term v, and the constant term 1 are combined into an augmented vector $z={\left[{\left(v⊙v\right)}^{T},v^{T},1\right]}^{T}$, and $\lambda =v^{T}v$ is defined. Under this definition, Equations (29)–(31) can be uniformly rearranged as a linear mapping of the augmented vector z, yielding

(32) Mz=λz,

where M is a 5 × 5 symmetric matrix defined as

(33) $$M=\left[\begin{array}{ccc} -k_{4}^{\prime }/k_{1}\; & -\mathrm{diag}(k_{5}^{\prime }/k_{1}\;) & 0_{2\times 1}\\ 0_{2\times 2} & -k_{4}^{\prime }/k_{1}\; & -k_{5}^{\prime }/k_{1}\;\\ 1_{2}^{T} & 0_{1\times 2} & 0 \end{array}\right],$$

where $0_{j\times k}$ denotes a j×k zero matrix with all entries equal to zero. The λ and z satisfying Equation (32) are the eigenvalue and the corresponding eigenvector of matrix M, respectively. According to Equation (31), the eigenvalue λ is equal to the sum of the first two elements of the eigenvector z.

Performing eigenvalue decomposition on matrix M yields

(34) $$M=\mathrm{UD}U^{-1},$$

where D is the diagonal matrix composed of the eigenvalues $\lambda_{m}$ (m=1,⋯,5) of M, and U is the orthogonal matrix formed by the normalized eigenvectors $z_{m}$ of M. For each eigenvector $z_{m}$, the sum of its first two elements is generally not equal to the corresponding eigenvalue $\lambda_{m}$, thus requiring a scaling operation. After scaling, the third and fourth components of the eigenvector are taken as the estimate of v, which is then substituted into Equation (24) to obtain the potential target position estimate, given by

(35) $${\hat{u}}_{m}=\frac{\lambda_{m}}{e_{1,2}^{T}z_{m}}{\left[e_{3},e_{4}\right]}^{T}z_{m}+s_{0},$$

where $e_{j,k}$ denotes a five-dimensional column vector with ones in the *j*-th and *k*-th positions and zeros elsewhere. Similarly, $e_{j}$ is a five-dimensional column vector with one in the *j*-th position and zeros elsewhere. Based on the distinct eigenvalues $\lambda_{m}$ and their corresponding normalized eigenvectors $z_{m}$, five potential position estimates ${\hat{u}}_{m}$ can be obtained. These estimates are then sequentially substituted into Equation (7) to evaluate the GLS cost function $J_{1}\left(u\right)$. The ${\hat{u}}_{m}$ that minimizes $J_{1}\left(u\right)$ is selected as the final target position estimate $\hat{u}$.

## 4. Estimation of the Path-Loss Exponent

In Section 3, the target position estimate is obtained under a preset path-loss exponent η. Since a deviation exists between the preset η and its true value, the path-loss exponent must be estimated. Using Equation (4) as the differential model, the corresponding GLS cost function has a form analogous to that in Equation (7), with the optimization variable changed from the target position vector u to the path-loss exponent η, i.e.,

(36) $$J_{3}\left(\eta \right)=g^{T}C_{\varepsilon }^{-1}g.$$

By minimizing the cost function $J_{3}\left(\eta \right)$, the GLS estimate of the path-loss exponent η is obtained as

(37) $$\hat{\eta }=\frac{\sum_{n=2}^{N}\left[\left(\mathrm{lg}d_{1}-\mathrm{lg}d_{n}\right)\left(r_{n}-r_{1}\right)\right]-\frac{1}{N}\sum_{n=2}^{N}\left(\mathrm{lg}d_{1}-\mathrm{lg}d_{n}\right)\sum_{n=2}^{N}\left(r_{n}-r_{1}\right)}{10\left\{\sum_{n=2}^{N}{\left(\mathrm{lg}d_{1}-\mathrm{lg}d_{n}\right)}^{2}-\frac{1}{N}{\left[\sum_{n=2}^{N}\left(\mathrm{lg}d_{1}-\mathrm{lg}d_{n}\right)\right]}^{2}\right\}}.$$

By substituting the estimated path-loss exponent $\hat{\eta }$ back into the differential model in Equation (4) and repeating the estimation procedure in Section 3, an improved target position estimate $\hat{u}$ can be obtained.

## 5. DIJE Method

As previously discussed, based on the differential model in Equation (4), a coarse estimate of the target position $\hat{u}$ can be obtained under a preset path-loss exponent η. Therefore, an iterative updating approach is adopted, taking RSSI measurements, AP coordinates, and a preset path-loss exponent as inputs. The target position and path-loss exponent are sequentially estimated and updated until convergence is achieved. Based on this procedure, a Differential Iterative Joint Estimation Approach for the two parameters, referred to as the DIJE method, is established. Building on the preliminary derivations in the preceding sections, this section presents a detailed introduction to the DIJE method.

The specific procedure of the DIJE method is summarized in Algorithm 1, which is divided into five main stages: constructing matrix M, estimating the target position, estimating the path-loss exponent, calculating the postiteration cost function value, and defining the iteration termination criterion.

Steps 1–5 in Algorithm 1 are dedicated to constructing matrix M. Given the input RSSI measurements ${\left\{r_{n}\right\}}_{n=1}^{N}$ and the preset path-loss exponent η, the translation vector $s_{0}$ between the original and new coordinate systems is first determined. Matrix M is then constructed using the coefficients $k_{4}^{\prime }$ and $k_{5}^{\prime }$ in the new coordinate system.

The second stage of the DIJE method is target position estimation, implemented in Steps 6–8. In this stage, the potential target position estimates ${\left\{{\hat{u}}_{m}\right\}}_{m=1}^{5}$ are obtained from the eigenvalues ${\left\{\lambda_{m}\right\}}_{m=1}^{5}$ and normalized eigenvectors ${\left\{z_{m}\right\}}_{m=1}^{5}$ of matrix M. The estimate ${\hat{u}}_{m}$ that minimizes the GLS cost function $J_{1}\left(u\right)$ is selected as the target position estimate $\hat{u}$.

Steps 9–10 are devoted to path-loss exponent estimation. In this stage, with the target position estimate $\hat{u}$ treated as a known quantity, the GLS estimate $\hat{\eta }$ of the path-loss exponent is derived from the differential model in Equation (4).

Steps 11–12 detail the calculation of the postiteration cost function value $J_{\mathrm{Iter}}$ after completing one iteration. The final stage of the DIJE method, specified in Step 13, defines the iteration termination logic. Upon convergence, the final target position estimate is taken as the ultimate target position estimate $\hat{u}$.
**Algorithm 1** DIJE method for indoor target localization**Input:** The position vectors ${\left\{s_{n}\right\}}_{n=1}^{N}$ of each AP in the WSN, the RSSI measurements ${\left\{r_{n}\right\}}_{n=1}^{N}$ received from the target, and the preset path-loss exponent η **Output:** The target position estimate $\hat{u}$      ∖∗Constructing the matrix M∗∖
  1:Given $p_{n}=\exp\left(k\cdot r_{n}\right)$, compute ${\left\{p_{n}\right\}}_{n=1}^{N}$ using the input ${\left\{r_{n}\right\}}_{n=1}^{N}$ and η, then calculate the coefficients ${\left\{a_{n}\right\}}_{n=1}^{N}$, ${\left\{b_{n}\right\}}_{n=1}^{N}$ and ${\left\{c_{n}\right\}}_{n=1}^{N}$;  2:Compute $k_{1}$ and $k_{2}$ from Equations (19) and (20);  3:Determine the translation vector $s_{0}$ between the original and new coordinate systems using $k_{1}$ and $k_{2}$;  4:Express the position vectors of all APs in the new coordinate system via $s_{0}$, update ${\left\{b_{n}\right\}}_{n=1}^{N}$ and ${\left\{c_{n}\right\}}_{n=1}^{N}$ accordingly, and obtain the new coefficients $k_{4}^{\prime }$ and $k_{5}^{\prime }$;  5:Construct matrix M according to Equation (33);∖∗Estimating the target position∗∖  6:Perform eigenvalue decomposition on M to obtain the eigenvalues ${\left\{\lambda_{m}\right\}}_{m=1}^{5}$ and the normalized eigenvectors ${\left\{z_{m}\right\}}_{m=1}^{5}$;  7:Substitute ${\left\{\lambda_{m}\right\}}_{m=1}^{5}$ and their corresponding ${\left\{z_{m}\right\}}_{m=1}^{5}$ into Equation (35) to compute the potential target position estimates ${\left\{{\hat{u}}_{m}\right\}}_{m=1}^{5}$;  8:Substitute each ${\hat{u}}_{m}$ into Equation (7), and select the ${\hat{u}}_{m}$ that minimizes the GLS cost function $J_{1}\left(u\right)$ as the target position estimate $\hat{u}$;∖∗Estimating the path-loss exponent∗∖  9:Compute the distances ${\left\{d_{n}\right\}}_{n=1}^{N}$ between the target and each AP using the target position estimate $\hat{u}$;10:Substitute ${\left\{r_{n}\right\}}_{n=1}^{N}$ and ${\left\{d_{n}\right\}}_{n=1}^{N}$ into Equation (37) to calculate the path-loss exponent estimate $\hat{\eta }$;∖∗Calculating the postiteration cost function value∗∖11:Substitute $\hat{u}$ and $\hat{\eta }$ into Equation (6) to compute the residuals ${\left\{g_{n}\right\}}_{n=2}^{N}$;12:Construct the residual vector from ${\left\{g_{n}\right\}}_{n=2}^{N}$, compute the GLS cost function $J_{1}\left(u\right)$ via Equation (7), and record this value as the postiteration cost, denoted $J_{\mathrm{Iter}}$;∖∗Iteration termination criterion∗∖13:Repeat the above steps until either the absolute or relative difference between two successive iterations is below a predefined threshold, or the number of iterations reaches the preset maximum. Terminate the iteration and output the final target position estimate $\hat{u}$.


## 6. Analysis of the Theoretical Optimal Lower Bound

Based on the differential model in Equation (4), the DIJE method employs an iterative scheme to jointly estimate the target position u and path-loss exponent η in the model. To evaluate the parameter estimation performance of the DIJE method, it is necessary to derive the CRLB for this differential model.

Define the vector of unknown parameters to be estimated as $\theta ={\left[x,y,\eta \right]}^{T}$. Then, the log-likelihood function of the differential model is given by

(38) $$L\left(\theta \right)=c-\frac{1}{2}g^{T}C_{\varepsilon }^{-1}g,$$

where *c* is a constant term. Therefore, the Fisher Information Matrix (FIM) can be expressed as

(39) $$F\left(\theta \right)=E\left[\frac{\partial L(\theta )}{\partial \theta }{\left(\frac{\partial L(\theta )}{\partial \theta }\right)}^{T}\right]=E\left[{\left(\frac{\partial g}{\partial \theta }\right)}^{T}C_{\varepsilon }^{-1}gg^{T}C_{\varepsilon }^{-1}\frac{\partial g}{\partial \theta }\right],$$

where

(40) $$\frac{\partial g}{\partial \theta }=\left[\frac{\partial g}{\partial x},\frac{\partial g}{\partial y},\frac{\partial g}{\partial \eta }\right],$$

with the partial derivatives given by

(41) $$\frac{\partial g}{\partial x}=2\frac{g_{x}}{k}-2\frac{x-x_{1}}{kd_{1}^{2}}1_{N-1},$$

(42) $$\frac{\partial g}{\partial y}=2\frac{g_{y}}{k}-2\frac{y-y_{1}}{kd_{1}^{2}}1_{N-1},$$

(43) $$\frac{\partial g}{\partial \eta }=\frac{5}{\ln10}g_{\eta }-\frac{5}{\ln10}\ln d_{1}^{2}1_{N-1}.$$

Here, $g_{x}={\left[\frac{x-x_{2}}{d_{2}^{2}},\cdots ,\frac{x-x_{n}}{d_{n}^{2}}\right]}^{T}$, $g_{y}={\left[\frac{y-y_{2}}{d_{2}^{2}},\cdots ,\frac{y-y_{n}}{d_{n}^{2}}\right]}^{T}$ and $g_{\eta }={\left[\ln d_{2}^{2},\cdots ,\ln d_{n}^{2}\right]}^{T}$.

Based on the FIM defined in Equation (39), the corresponding CRLB is given by

(44) $$\mathrm{CRLB}(\theta_{i})={\left[F^{-1}\left(\theta \right)\right]}_{ii}.$$

Thus, the CRLB for the target position estimation is

(45) $$\mathrm{CRLB}(u)={\left[F^{-1}\left(\theta \right)\right]}_{11}+{\left[F^{-1}\left(\theta \right)\right]}_{22},$$

and the CRLB for the path-loss exponent estimation is

(46) $$\mathrm{CRLB}(\eta )={\left[F^{-1}\left(\theta \right)\right]}_{33}.$$

## 7. Simulation Results

### 7.1. Localization Performance of the DIJE Method

To comprehensively evaluate the localization robustness of the DIJE method under parameter uncertainty, simulation experiments are conducted to analyze the impact of shadow fading on positioning accuracy under different initial path-loss exponent conditions. The simulation setup is consistent with the baseline conditions in Section 2.3, with only the key parameters adjusted: the AP deployment radius is uniformly set to 15 m, the standard deviation σ of shadow fading ranges from 0.5 to 3 dB, and the initial path-loss exponent $\eta_{0}$ is selected as five groups of typical values around the true value η = 4 (i.e., $\eta_{0}$ = 3, 3.8, 4, 4.2, 5), covering the three scenarios of initial value bias: underestimated, neartrue, and overestimated. All experiments are carried out in a gridded localization area, and 90,000 Monte Carlo simulations are performed for each parameter combination to eliminate the influence of random errors on the results. The maximum number of iterations is set to 30. The iteration is terminated when the absolute difference between two consecutive iteration results is smaller than $10^{-3}$, the relative difference is smaller than $10^{-4}$, or the maximum number of iterations is reached. All experiments are conducted on a Windows 10 platform with an Intel Core i7-10700 CPU and 16 GB RAM. All algorithms are implemented in MATLAB R2022b without GPU acceleration or parallel computing.

**Figure 3:**
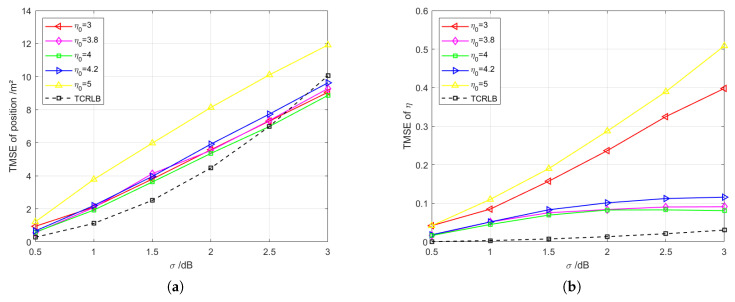
TMSE performance versus shadow fading standard deviation σ under different initial path-loss exponent conditions: (**a**) target position TMSE; (**b**) path-loss exponent estimation TMSE.

Based on the simulation results, the total mean square error of the target position ${\mathrm{TMSE}}_{p}$ and the total mean square error of the path-loss exponent estimation ${\mathrm{TMSE}}_{\eta }$ are calculated, both defined in the same manner as Equation (8). The algorithm errors are compared with the Total CRLB (TCRLB) of the corresponding scenarios, and the experimental results are presented in Figure 3. The TCRLB is defined as the arithmetic mean of the CRLBs at all localization points in the positioning scenario.

As observed from Figure 3, an increase in the shadow fading standard deviation σ induces a systematic degradation in both localization and parameter estimation performance. The target position ${\mathrm{TMSE}}_{p}$ and path-loss exponent estimation ${\mathrm{TMSE}}_{\eta }$ both increase monotonically with σ, indicating that elevated noise intensity simultaneously degrades the target localization accuracy and model parameter estimation accuracy. The theoretical lower bound TCRLB also rises synchronously with σ, demonstrating that increased noise levels raise the theoretical performance limit of the estimation problem. The growth in algorithm error is fundamentally attributed to the reduction in the information entropy of the problem itself, rather than algorithmic deficiencies.

As observed from the target localization error curves in Figure 3a, the localization error increases monotonically with noise intensity across all initial-value scenarios, while the magnitude of degradation varies significantly for different initial values. When $\eta_{0}$ = 5, the localization error remains consistently higher than that of all other groups, with the steepest degradation rate as σ increases. This indicates that an excessively large initial bias leads to irreversible deterioration in localization performance. Comparing Figure 3a with Figure 2 in Section 2.3, it can be seen that in Section 2.3, path-loss exponent mismatch causes a significant amplification of localization error, whereas, in this section, the DIJE method exhibits only minor differences in localization error under different initial values. This demonstrates that the algorithm can adaptively correct the path-loss exponent via the iterative process, effectively mitigating the impact of initial bias on localization performance and achieving strong robustness to initial values. It can also be seen from Figure 3a that the robustness of the DIJE method to initial values has a clear boundary. When the deviation between $\eta_{0}$ and the true value η = 4 is within ±0.2, the localization performance is nearly unaffected. When the deviation increases to ±1, the degradation in localization performance becomes directional: specifically, when $\eta_{0}$ = 3, the localization performance degrades only slightly, while when $\eta_{0}$ = 5, it deteriorates significantly. This verifies the practical applicability of the proposed algorithm in engineering applications.

As observed from the path-loss exponent error curves in Figure 3b, the estimation of the path-loss exponent is significantly more sensitive to the initial value than target localization, exhibiting a distinct stratification characteristic. When $\eta_{0}$ = 4, ${\mathrm{TMSE}}_{\eta }$ remains at the minimum level across the entire noise range and increases smoothly, indicating that the DIJE method achieves high-accuracy parameter estimation when the initial value is accurate. When $\eta_{0}$ = 3.8 and $\eta_{0}$ = 4.2 (i.e., the deviation between the initial value and the true value is ±0.2), the error curves nearly overlap with that for $\eta_{0}$ = 4, demonstrating that the algorithm maintains stable parameter estimation accuracy under small perturbations around the true value. When $\eta_{0}$ = 3 and $\eta_{0}$ = 5 (i.e., the deviation between the initial value and the true value is ±1), the parameter estimation error increases significantly and grows approximately exponentially with σ. Furthermore, the degradation magnitude for $\eta_{0}$ = 5 is substantially larger than that for $\eta_{0}$ = 3, indicating that an excessively large initial value exerts a far stronger negative impact on parameter estimation than an excessively small one. Although parameter estimation is highly sensitive to the initial value, the target localization performance presented in Figure 3a still retains strong robustness. This phenomenon reveals a core advantage of the DIJE method: the algorithm implements an adaptive mechanism via iterative optimization that prioritizes localization accuracy. Even when the parameter estimation contains bias, the localization result can still be corrected through iteration to guarantee its accuracy, thereby addressing the key drawback of conventional localization algorithms—localization failure caused by parameter mismatch.

Based on the results presented in Figure 3a,b, the DIJE method demonstrates stable localization performance across different noise intensities and initial path-loss exponent conditions. The position estimation exhibits extremely high robustness to the initial value of the path-loss exponent: even when the initial value deviates to a certain extent, the DIJE method maintains a low localization error via iterative updates. While the path-loss exponent estimation is more sensitive to the initial value, this sensitivity only affects the accuracy of the path-loss exponent estimate and does not cause significant degradation to the localization performance, reflecting the core optimization objective of the DIJE method in localization tasks. In scenarios where the initial path-loss exponent is within 5% of the true value, the position estimation error of the DIJE method differs from the TCRLB by less than 2 m^2^, and the path-loss exponent estimation error deviates from the TCRLB by less than 0.1. This demonstrates that the proposed algorithm can achieve favorable localization performance under parameter-known scenarios, thereby verifying its theoretical soundness.

In summary, the DIJE method is robust to parameter initialization errors, effectively mitigating the impact of incorrect initial path-loss exponent values. It achieves accurate and stable target localization in various noise environments, satisfying the positioning requirements for complex scenarios.

### 7.2. Effect of the Initial Path-Loss Exponent on Localization Accuracy

**Figure 4:**
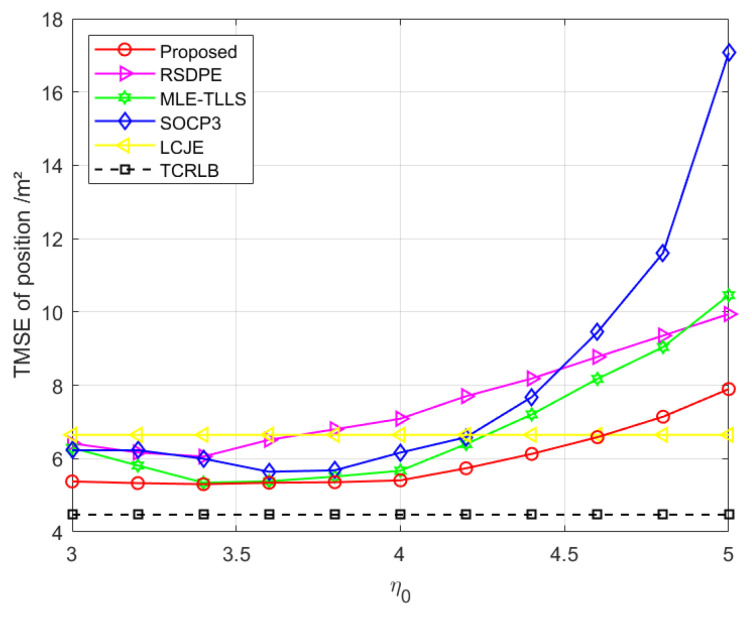
Position estimation TMSE versus initial path-loss exponent $\eta_{0}$ for different algorithms, with σ = 2 dB.

To systematically evaluate the localization performance of the proposed DIJE method under uncertainty in the initial path-loss exponent and verify its robustness to initial value selection, the following simulation experiments are designed. The experimental setup follows the basic configuration in Section 7.1, with the shadow fading standard deviation fixed at the typical value σ = 2 dB. The proposed DIJE method is compared with SOCP3 [16], RSDPE [17], MLE-TLLS [18], LCJE [19], and the theoretical lower bound TCRLB. Among these, the DIJE, RSDPE, MLE-TLLS, and SOCP3 algorithms require a preset initial path-loss exponent during the solution process, whereas the LCJE algorithm does not depend on this initial value. The variation of the total mean square error of position estimation ${\mathrm{TMSE}}_{p}$ with the initial path-loss exponent $\eta_{0}$ for different algorithms is presented in Figure 4.

As observed from Figure 4, under uncertainty in the path-loss exponent, the TMSE of position estimation for each algorithm exhibits significantly distinct evolutionary trends as the initial value varies, directly reflecting the fundamental differences in sensitivity to initial values among different methods. The proposed DIJE method maintains an extremely low localization error throughout the entire interval $\eta_{0}\in$ [3, 5], with minimal fluctuations in the error curve. When $\eta_{0}$ lies within [3, 4], the ${\mathrm{TMSE}}_{p}$ of the algorithm stabilizes in the range of 5.2–6 m^2^, with only a slight increase as $\eta_{0}$ approaches 5, and the final error remains below 8 m^2^. This fully demonstrates its strong robustness to the initial path-loss exponent.

Comparing the performance differences among the algorithms, the MLE-TLLS method achieves localization accuracy comparable to that of the proposed DIJE method when $\eta_{0}$ is close to the true value, with relatively mild fluctuations in error as the initial value varies. In contrast, the RSDPE algorithm exhibits a distinct error behavior: its ${\mathrm{TMSE}}_{p}$ shows a gradual upward trend as $\eta_{0}$ deviates from the true value, and the consistency of its localization accuracy is significantly inferior to that of the proposed method. Neither algorithm is free from the constraint of initial value sensitivity. As $\eta_{0}$ continues to increase, the ${\mathrm{TMSE}}_{p}$ of both RSDPE and MLE-TLLS shows a monotonically increasing trend, rising to 9.9 m^2^ and 10.4 m^2^ respectively at $\eta_{0}$ = 5, resulting in a clear degradation in localization performance. Overall, the error levels of these two algorithms are significantly higher than those of the proposed DIJE method.

The SOCP3 algorithm exhibits a typical error evolution characterized by stability under low initial values and divergence under high initial values. Within the interval $\eta_{0}\in$ [3, 4.2], its ${\mathrm{TMSE}}_{p}$ remains at a low level of 6–6.5 m^2^. However, once $\eta_{0}$ exceeds 4.2, the error increases sharply and exponentially, reaching 17.1 m^2^ at $\eta_{0}$ = 5. This indicates that the algorithm is highly sensitive to large initial value mismatches and exhibits poor robustness. The LCJE algorithm maintains a flat error curve across the entire interval, which stems from its inherent property of not requiring a preset initial path-loss exponent. Nevertheless, its overall error level consistently exceeds 6.7 m^2^, which is significantly inferior to that of the proposed DIJE method, confirming its inherent limitations in localization accuracy.

Further combining the analysis with the theoretical performance lower bound TCRLB, the theoretical reference lower bound under the present experimental conditions is $\mathrm{TCRLB}=4.5\;m^{2}$. The minimum position estimation error of the proposed DIJE method occurs at $\eta_{0}=4$, with ${\mathrm{TMSE}}_{p}=5.2\;m^{2}$. The absolute gap between DIJE and the TCRLB is approximately 0.7 m^2^, corresponding to a relative gap of about 15%. Therefore, DIJE does not strictly attain the theoretical lower bound, but its error remains close to the TCRLB with a small and quantifiable gap. Over the entire initial-value range $\eta_{0}\in \left[3,5\right]$, the ${\mathrm{TMSE}}_{p}$ of DIJE exhibits only limited variation and remains the closest to the TCRLB among all compared algorithms. These results indicate that although DIJE does not exactly reach the theoretical lower bound, it achieves the closest overall error level to the TCRLB under the current experimental conditions, while also demonstrating superior robustness to the initial value and better overall localization performance than the existing comparison methods.

To further validate the above results from the perspectives of statistical stability and computational efficiency, we calculate the average single-positioning time and the 95% confidence interval of ${\mathrm{TMSE}}_{p}$ under the initial condition $\eta_{0}=4$. The results are shown in Table 2.

As shown in Table 2, under the conditions of $\eta_{0}=4$, the 95% confidence interval of the proposed DIJE method’s positioning error is 0.24 $m^{2}$, which is closer to MLE-TLLS’s 0.22 $m^{2}$. It is also notably smaller than RSDPE’s 0.76 $m^{2}$, SOCP3’s 2.35 $m^{2}$, and LCJE’s 0.35 $m^{2}$. This demonstrates that the DIJE method exhibits relatively low positioning variance and maintains good statistical stability. Its performance advantage is primarily due to the experimental results, where the larger variance observed for SOCP3 indicates a broader confidence region and poor stability under such parameters.

From the perspective of computational efficiency, the average single-positioning time of DIJE is 2.44 ms, which is on the same order as that of LCJE, and is significantly lower than those of MLE-TLLS and RSDPE. The average runtime of SOCP3 reaches 4352 ms, which is much higher than that of the other algorithms. This indicates that although relaxation-based optimization methods can provide a certain level of localization accuracy, their computational cost is relatively high, making them difficult to satisfy low-latency localization requirements. Overall, DIJE maintains low positioning error and a narrow confidence interval while still achieving millisecond-level computational efficiency, demonstrating a favorable balance among accuracy, stability, and computational complexity.

In summary, the proposed DIJE method consistently achieves lower localization errors and more stable performance under different initial path-loss exponent conditions, fully demonstrating its superior adaptability to initial values and excellent comprehensive localization performance in scenarios with inaccurate parameters. A deeper analysis reveals that the performance advantage of DIJE does not arise from a simple combination or local improvement of existing joint estimation methods, but from its systematic differences in estimation mechanisms. Specifically, DIJE first utilizes a differential model to analytically eliminate the influence of reference RSSI, thereby reducing the coupling between reference RSSI, path-loss exponent, and target location. Building on this, a fast position estimation is achieved under smaller approximation conditions through an eigenvalue decomposition mechanism, which avoids the computational burden of large-scale convex relaxation or high-dimensional nonlinear search. At the same time, the iterative update of the path-loss exponent allows for adaptive joint correction of propagation parameters and target position. Therefore, DIJE is able to maintain stable and high positioning accuracy across a wide range of initial parameter settings, demonstrating superior overall performance compared to existing joint estimation methods such as RSDPE, LCJE, and MLE-TLLS.

### 7.3. Effect of the Number of APs on Algorithm Performance

**Figure 5:**
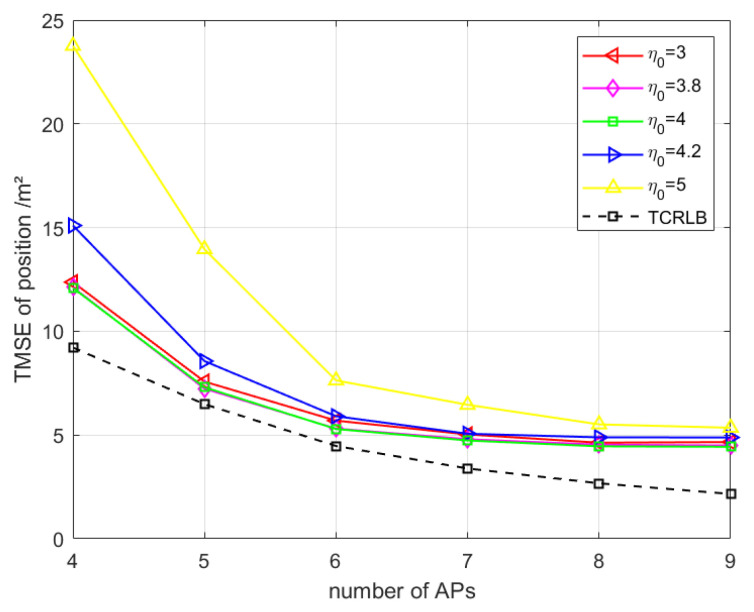
The TMSE of target position versus the number of access points under different initial path-loss exponent settings.

To comprehensively evaluate the localization performance of the proposed DIJE method under different network deployment densities, and to further quantify the robustness of the algorithm to the initial bias of the path-loss exponent in terms of positioning accuracy, this section conducts a comparative analysis by adjusting key parameters based on the simulation experimental framework presented in Section 7.1. The number of APs is set to range from 4 to 9, the shadow fading standard deviation σ is fixed at 2 dB, and all other experimental scenarios and parameter settings remain consistent. Based on the simulation results, the total mean square error of the target position ${\mathrm{TMSE}}_{p}$ is statistically calculated, and compared with the TCRLB under the corresponding scenarios, with the results illustrated in Figure 5.

As illustrated in Figure 5, both the localization error of the proposed DIJE method and the TCRLB exhibit a strictly monotonic decreasing trend as the number of APs increases. This verifies, from both experimental and theoretical perspectives, the positive performance gain of increasing network deployment density for positioning systems. Specifically, the reduction in localization error demonstrates a distinct two-stage characteristic. The first stage is a rapid decline phase when the number of APs increases from 4 to 6. Taking the initial path-loss exponent setting $\eta_{0}=4$ as an example, the TMSE of the target position ${\mathrm{TMSE}}_{p}$ drops sharply from approximately 12.5 m^2^ to 5.2 m^2^, representing a reduction of over 60%. This indicates that in the low-density deployment regime, increasing the number of APs enables a significant improvement in localization performance at a relatively low cost. The second stage is a slow convergence phase when the number of APs increases from 6 to 9, where the error reduction rate slows down substantially and eventually stabilizes. This reveals that the localization gain brought by increasing the number of nodes exhibits a clear diminishing marginal effect. Consequently, in practical engineering deployment, a trade-off between performance enhancement and hardware cost is required to achieve optimal cost-effectiveness.

From the comparative results under different initial path-loss exponent settings, it can be observed that the number of APs exerts a significant regulatory effect on the robustness of the proposed DIJE method, exhibiting a distinct hierarchical convergence characteristic. In low-density deployment scenarios with a limited number of APs, the localization error curves corresponding to different initial path-loss exponent values show pronounced discrepancies. Specifically, the localization error for the initial value $\eta_{0}$ = 5 (severely deviating from the true value η = 4) is substantially higher than that of all other groups, followed by $\eta_{0}$ = 3 and $\eta_{0}$ = 4.2, while the curves for $\eta_{0}$ = 3.8 and $\eta_{0}$ = 4 (close to the true value) nearly coincide. This indicates that in low-density deployment regimes, initial value deviations are amplified, leading to irreversible degradation in localization performance. Moreover, the negative impact of an overestimated initial value is significantly stronger than that of an underestimated one, demonstrating a clear asymmetric characteristic. As the number of APs continues to increase, the error curves for different initial path-loss exponent values gradually converge and become consistent. When the number of APs exceeds six, the localization errors for all initial values enter a stable convergence state: the four curves for $\eta_{0}$ = 3, 3.8, 4, 4.2 fully coincide, while the error for $\eta_{0}$ = 5 remains slightly higher than the others, yet the gap is substantially narrowed and remains stable without further degradation. This result fully verifies that a higher network deployment density can effectively mitigate the impact of initial value deviations on localization performance. The underlying mechanism is that the participation of more APs provides richer spatial observation information, enhancing the ability of the localization model to suppress parameter mismatches. This enables the algorithm to counteract initial deviations through iterative correction, thereby significantly improving the stability and robustness of localization. Furthermore, once the deployment density reaches a certain threshold (i.e., when the number of APs exceeds six), the robustness of the DIJE method to initial values enters a saturated state, and localization performance no longer improves significantly with further increases in the number of APs.

Further comparative analysis between the localization error of the proposed DIJE method and the TCRLB reveals that both quantities exhibit consistent, strictly monotonic decreasing trends as the number of APs increases. This theoretically verifies the positive performance gain of increasing network deployment density for positioning systems. Specifically, in scenarios where the initial path-loss exponent is close to the true value (i.e., $\eta_{0}$ = 3.8, 4, 4.2), the gap between the DIJE method’s localization error and the TCRLB demonstrates a distinct two-stage characteristic: an initial narrowing followed by a subsequent widening. As the number of APs increases from four to six, the discrepancy between the algorithm’s error and the theoretical lower bound continuously diminishes, reaching its minimum at six APs. When the number of APs exceeds six, this gap progressively widens with further increases in AP count, with the algorithm’s localization error consistently remaining above the TCRLB and the deviation magnitude growing gradually. For scenarios with severe initial value deviations (i.e., $\eta_{0}$ = 3, 5), the DIJE method’s localization error is significantly higher than the TCRLB, with the gap for $\eta_{0}$ = 5 being the largest across the entire range of AP numbers. The gaps for these two severe deviation scenarios also follow the same pattern: an initial narrowing, followed by a continuous widening as the number of APs increases, and the larger the initial deviation, the more pronounced the deviation at high AP densities. This phenomenon indicates that although the algorithm’s localization error continues to decrease and eventually stabilizes with an increasing number of APs, the gap between the algorithm’s actual performance and the theoretical optimal performance gradually widens in high-density deployment scenarios. The underlying mechanism is that as the redundancy of observational information increases, the impact of the iterative correction error of the path-loss exponent on the localization result is amplified, making it difficult for the algorithm to further approach the theoretical lower bound.

In conclusion, the proposed DIJE method demonstrates excellent localization performance across all tested network deployment densities. Specifically, in low-to-medium density deployment scenarios, the method achieves positioning accuracy approaching the theoretical optimum. In high-density deployment scenarios, the algorithm maintains exceptional robustness against deviations in the initial path-loss exponent, with localization errors stabilizing at consistently low levels. These attributes enable the DIJE method to effectively adapt to complex and dynamic real-world deployment environments, thereby providing a reliable technical foundation for the engineering implementation of wireless localization systems.

## 8. Experimental Results on Public Datasets

To verify the localization accuracy and robustness of the proposed DIJE method in practical scenarios, this section conducts experimental validation in two typical indoor environments: a simple unobstructed indoor space and a complex indoor environment with obstacles. Through comparative analysis with classical benchmark algorithms, the localization performance of the DIJE method under real-world channel conditions is comprehensively evaluated.

### 8.1. Simple Indoor Scenario

**Figure 6:**
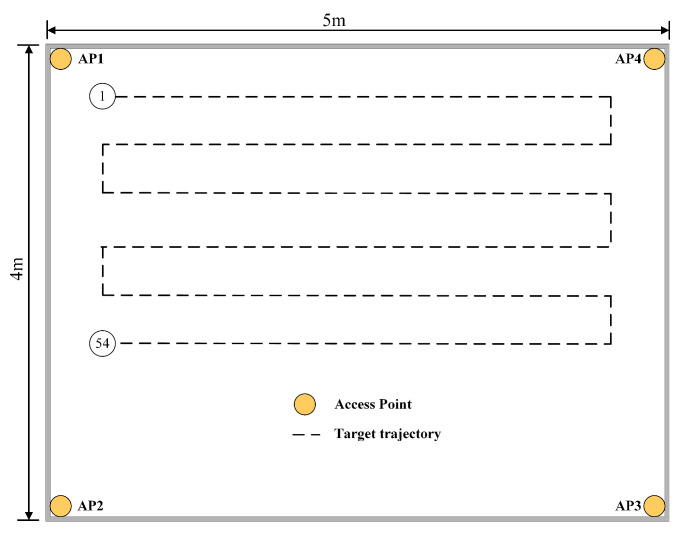
Experimental scenario and target trajectory in the simple indoor environment public dataset.

The experiments are conducted using the public dataset from Kadir Has University, Istanbul, Turkey [23]. This dataset was collected in a standardized indoor test platform with dimensions of 4 m × 5 m, and the experimental scenario is illustrated in Figure 6. Four APs are deployed at the four corners of the test area, and the target positions are uniformly arranged at an interval of 0.5 m, resulting in a total of 54 test sampling points. The logarithmic path-loss model is applied to fit the measured data via the least squares method, yielding the true path-loss exponent η = 1.9 and the reference RSSI value $r_{0}$ = −34.1 dBm for this scenario, thereby providing a benchmark of real channel parameters for subsequent algorithm validation.

In the comparative algorithm experiments, for the proposed DIJE method, the initial path-loss exponent is set to $\eta_{0}\in$ {1.2, 1.9, 2.5} to conduct localization tests and analyze the impact of initial parameters on the algorithm’s localization performance. Two classical localization algorithms are selected as comparison benchmarks: RSDPE [17] based on semidefinite joint estimation and MLE-TLLS [18] based on the maximum likelihood frame-work. For both benchmark algorithms, the initial path-loss exponent is uniformly set to $\eta_{0}$ = 1.5 to simulate scenarios where the initial parameter deviates from the true value in practical applications. Furthermore, the ANLS algorithm [24], which assumes perfect knowledge of the true path-loss exponent, is introduced as an upper performance bound. In one set of experiments, the ANLS algorithm uses the true path-loss exponent $\eta_{0}$ = 1.9 for localization; in another set, a biased path-loss exponent $\eta_{0}$ = 1.5 is adopted to simulate the impact of model parameter errors on localization accuracy in practical applications, thereby verifying the robustness of the proposed joint estimation framework against initial value mismatches.

**Figure 7:**
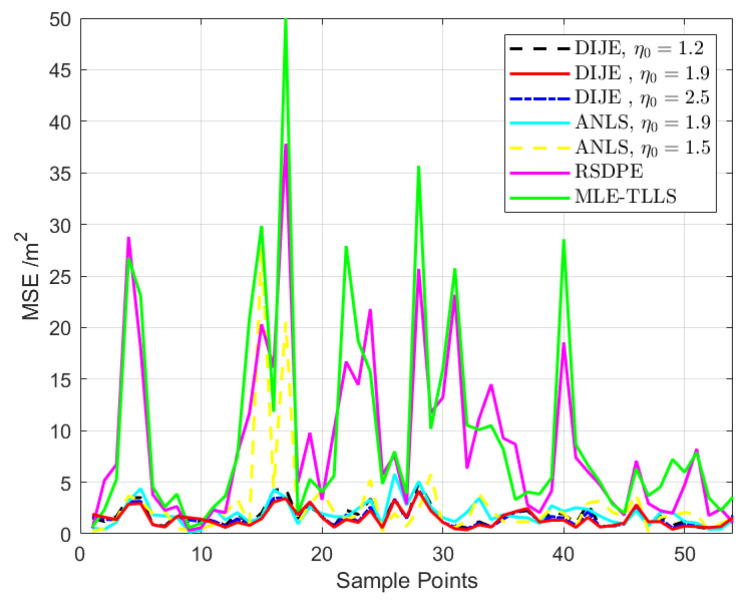
Comparison of the ${\mathrm{MSE}}_{p}$ distribution for different algorithms under various initial path-loss exponent conditions on the public dataset.

The distribution of the total mean square error of position ${\mathrm{MSE}}_{p}$ across all 54 test points, obtained by different algorithms under various initial path-loss exponent conditions, is presented in Figure 7.

Table 3 reports the ${\mathrm{TMSE}}_{p}$ for each method, providing a quantitative basis for comparing the overall localization accuracy.

From the perspective of the overall error distribution, the proposed DIJE method maintains consistently low and stable localization errors at the vast majority of test points. As shown in Table 3, regardless of the initial value conditions, the DIJE method outperforms both the RSDPE and MLE-TLLS algorithms in terms of overall localization accuracy. Specifically, the error curves of the RSDPE and MLE-TLLS algorithms exhibit substantial fluctuations, with the ${\mathrm{MSE}}_{p}$ values at certain test points showing large abrupt spikes, reaching a maximum error of 50 m^2^. This indicates that traditional fixed-parameter or single-parameter estimation methods suffer from evident stability deficiencies, even in simple, unobstructed real-world indoor environments. In contrast, the error curve of the DIJE method is generally smooth, with extremely small fluctuations across all test points, thereby validating the high stability of the proposed algorithm in real-world localization scenarios.

Regarding the localization performance of the proposed DIJE method under different initial path-loss exponent settings ($\eta_{0}$ = 1.2, 1.9, 2.5), the three error curves are nearly identical, with only minor discrepancies observed at a small number of test points. This demonstrates the extremely high robustness of the proposed method to initial parameter deviations: regardless of whether the initial value is lower than, equal to, or higher than the true path-loss exponent, the DIJE method converges to a near-optimal localization result via iterative optimization, effectively overcoming the performance degradation caused by initial parameter mismatches. This fundamentally addresses the limitation of traditional algorithms that rely on accurate prior parameters, thereby providing superior accuracy assurance capability in practical localization scenarios.

In stark contrast, for the ANLS benchmark algorithm the following is true: when the true path-loss exponent ($\eta_{0}$ = 1.9) is adopted, the localization error of the ANLS algorithm remains consistently low, verifying its theoretical performance advantage under perfectly accurate model parameters. However, when the path-loss exponent is set to a biased value ($\eta_{0}$ = 1.5), the localization error of the ANLS algorithm increases significantly, accompanied by intensified fluctuations in the error curve. This fully demonstrates that traditional fixed-parameter methods exhibit an extremely strong dependence on the accuracy of model parameters. Once the parameters deviate from their true values, the localization performance deteriorates sharply, making it difficult to adapt to scenarios with dynamically varying parameters in practical indoor environments.

Based on the comprehensive experimental results obtained from the real-world indoor RSSI dataset, the proposed DIJE method achieves dual improvements in both localization accuracy and robustness. On the one hand, compared with traditional benchmark algorithms such as RSDPE and MLE-TLLS, the DIJE method attains lower localization errors at the majority of test points, with significantly enhanced error stability, thereby effectively mitigating the issue of large fluctuations in localization results in real-world environments. On the other hand, the DIJE method eliminates the reliance of traditional algorithms on accurate initial parameters, maintaining stable localization performance even in scenarios with uncertainty in the path-loss model parameters. This perfectly satisfies the application requirements of practical indoor localization, where accurate calibration of model parameters is often challenging.

In conclusion, the experimental validation conducted in the simple indoor environment fully demonstrates the effectiveness and robustness of the proposed DIJE method for practical localization applications, establishing a reliable technical foundation for high-precision localization in complex indoor scenarios.

### 8.2. Complex Indoor Scenario

**Figure 8:**
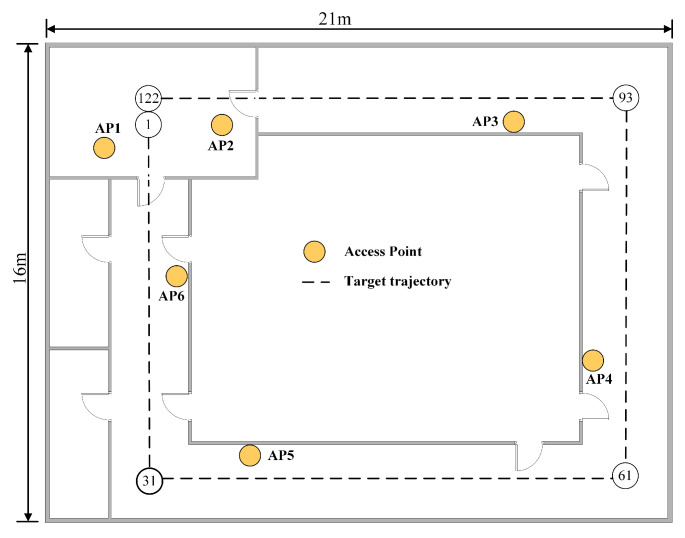
Experimental scenario and target trajectory in the complex indoor environment public dataset.

To further evaluate the robustness and engineering applicability of the proposed DIJE method in more complex real-world scenarios, this section conducts validation experiments using the public dataset provided in [25]. This dataset was collected in a complex indoor environment featuring a mixed structure of rooms and a long corridor, exhibiting significant multipath propagation effects and NLOS propagation characteristics. This establishes a stringent test benchmark for evaluating the localization performance of the DIJE method under non-ideal channel conditions. The layout of the experimental scenario is illustrated in Figure 8.

The experimental site measures 16 m × 21 m, comprising one independent room and three connected corridors. A total of six APs are deployed in the scenario, with coordinates given by $AP_{1}={\left[2,1.5\right]}^{T}$, $AP_{2}={\left[7,3\right]}^{T}$, $AP_{3}={\left[14.5,2\right]}^{T}$, $AP_{4}={\left[17,-7\right]}^{T}$, $AP_{5}={\left[10,-11\right]}^{T}$, $AP_{6}={\left[6,-6\right]}^{T}$. The target trajectory follows the dashed line illustrated in the figure, consisting of 122 discrete sampling points that cover diverse spatial regions, including the room interior and corridor passages. The collected RSSI data are fitted via the least squares method, yielding the true path-loss exponent η = 2.9 and the reference RSSI value $r_{0}$ = −32.5 dBm for this indoor environment, thereby providing accurate channel parameter benchmarks for algorithm performance evaluation.

This dataset exhibits two prominent typical characteristics. First, the target trajectory covers multiple types of areas, including the room interior and external corridors, demonstrating strong spatial heterogeneity. This enables a comprehensive assessment of the algorithm’s localization adaptability across different spatial locations. Second, the environment is characterized by complex multipath propagation effects. The heterogeneous structure of the room and corridors results in significantly distinct signal propagation characteristics, with more complex signal attenuation and reflection patterns, which poses a severe challenge to the algorithm’s anti-interference capability and parameter adaptability.

**Figure 9:**
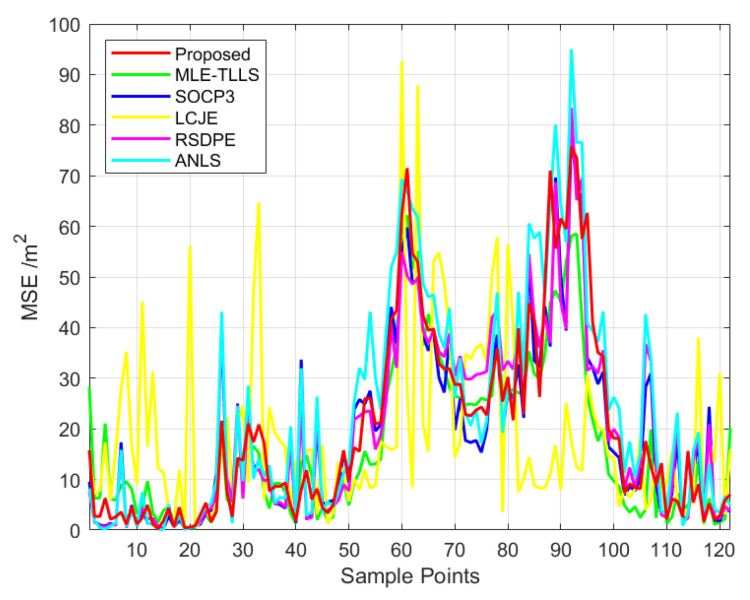
Comparison of ${\mathrm{MSE}}_{p}$ distributions for different algorithms on the real-world complex indoor environment dataset.

To comprehensively verify the performance advantages of the proposed DIJE method, five classical localization algorithms are selected as comparative benchmarks: the RSDPE method based on semidefinite joint estimation [17], the MLE-TLLS method based on the maximum likelihood framework [18], the SOCP3 algorithm based on second-order cone programming relaxation [16], the LCJE algorithm based on joint estimation under the maximum likelihood framework [19], and the ANLS method with the path-loss exponent fixed at $\eta_{0}$ = 2.5 [24]. Among these algorithms, the initial path-loss exponent $\eta_{0}$ for all joint estimation-based methods is uniformly set to two to simulate real-world scenarios where the initial model parameters deviate from their true values. By comparatively analyzing the ${\mathrm{MSE}}_{p}$ of each algorithm at different test points, the localization accuracy and robustness of the DIJE method are quantitatively evaluated. The ${\mathrm{MSE}}_{p}$ distributions of the different algorithms on this dataset are illustrated in Figure 9.

To provide a quantitative assessment of the localization performance, Table 4 presents the ${\mathrm{TMSE}}_{p}$ for each method, enabling a comparison of their overall accuracy.

As shown in Table 4, the DIJE method achieves superior overall localization accuracy compared to the other methods. From the overall trend of the error distribution in Figure 9, it can be observed that the proposed DIJE method achieves consistently low and stable localization errors at the vast majority of the 122 test sampling points. Its error curve is generally smooth, with fluctuation magnitudes significantly smaller than those of the other benchmark algorithms, fully demonstrating excellent localization stability and accuracy retention capability. In contrast, the other benchmark algorithms exhibit higher overall error levels and more severe curve fluctuations, with substantial increases in ${\mathrm{MSE}}_{p}$ values at certain test points. Among these, the ANLS method shows the most pronounced error fluctuations, with error values exceeding 80 m^2^ at multiple sampling points and peaking at nearly 100 m^2^. This indicates that traditional fixed-parameter algorithms struggle to adapt to the dynamic variations in signal propagation characteristics in complex multipath environments, revealing clear shortcomings in the reliability and stability of their localization results. Further analysis reveals that the core advantage of the DIJE method lies in its joint estimation framework, which simultaneously optimizes the path-loss exponent and target position parameters, enabling dynamic correction of model parameter deviations during the localization process. This mechanism fundamentally mitigates the interference of complex channel conditions on localization accuracy, thereby achieving dual guarantees of accuracy and stability throughout the experiments.

In certain segments of the target trajectory, such as the complex corridor structure area near sample points 60–90, the localization errors of all algorithms increase significantly. This phenomenon is highly consistent with the propagation characteristics of the complex channel environment. This region is adjacent to a corridor corner and a multistructure intersection, where multipath reflections and NLOS propagation are extremely pronounced. This leads to substantial distortions in the RSSI measurements and a significant reduction in the signal-to-noise ratio (SNR), thereby negatively impacting the localization accuracy of all algorithms. Even in this critical region where errors rise sharply, the proposed DIJE method exhibits a relatively small increase in error, with fluctuations in its error curve far smaller than those of the other benchmark algorithms. It maintains a relatively stable error level throughout, fully demonstrating its strong robustness in complex multipath propagation environments. In stark contrast, algorithms such as ANLS and LCJE experience a sharp surge in error in this region, with their error curves exhibiting severe fluctuations. This indicates that such algorithms have insufficient adaptability to complex channel environments, where model parameter deviations directly lead to significant degradation in localization performance.

For the MLE-TLLS, SOCP3, and RSDPE algorithms, although they achieve relatively low errors at some local test points, their overall mean errors are higher than that of the DIJE method. Furthermore, they exhibit pronounced error fluctuations at the majority of sampling points, making it difficult to achieve stable localization across the entire trajectory. This indicates that these three algorithms have clear limitations in terms of parameter estimation accuracy and anti-interference capability in complex heterogeneous environments. Although the LCJE algorithm achieves low error levels comparable to those of the DIJE method at a few test points, its overall error curve exhibits larger fluctuations. Consequently, the impact of model parameter deviations on its localization accuracy is more pronounced, leading to a sharp degradation in localization performance in high-error regions. This prevents stable localization over the entire trajectory. The ANLS method, as a representative of fixed-parameter algorithms, exhibits the worst overall performance. Not only are its error values consistently high, but the increase in error in complex channel regions is far more substantial than that of the other algorithms. This fully confirms the strong dependence of fixed-parameter methods on the accuracy of model parameters and their inability to adapt to dynamic parameter variations in practical complex indoor environments.

Based on the comprehensive experimental results obtained in the complex indoor environment, the proposed DIJE method demonstrates strong effectiveness and robustness in severe multipath and NLOS scenarios. Not only does it effectively reduce the overall localization error and achieve high-precision localization across the entire trajectory, but it also maintains a relatively small error growth amplitude and stable error performance in complex channel regions where errors are prone to sharp surges. This result validates the effectiveness and robustness of the DIJE method in complex real-world indoor environments. It demonstrates the capability of the proposed method to adapt to practical engineering scenarios involving complex indoor localization, thereby providing a practical and feasible technical solution for high-precision positioning in complex indoor environments.

## 9. Conclusions

This paper focuses on the indoor RSSI localization problem under reference RSSI value and path-loss exponent mismatch conditions, and proposes a DIJE localization method based on differential RSSI models and iterative estimation. The method establishes an alternating update mechanism for the target position and path-loss exponent, achieving adaptive joint estimation of model parameters and target positions, thereby fundamentally mitigating the performance degradation caused by severe model parameter mismatches. Specifically, in the position estimation module, the DIJE method employs a differential model to eliminate the uncertainty introduced by the reference RSSI value. By constructing an eigenvalue equation, it transforms the maximum likelihood estimation problem into an eigenvector solving problem, ensuring fast and high-accuracy target position estimation. In the path-loss exponent estimation module, the DIJE method leverages the linear structural characteristics of the model and adopts the generalized least squares method to efficiently solve for the parameters. This design balances estimation accuracy and computational efficiency, achieving a favorable trade-off between the two.

To comprehensively evaluate the comprehensive performance of the DIJE method, extensive experimental validations are conducted through simulations and public real-world dataset tests. The experimental results demonstrate that the proposed DIJE method exhibits stable localization performance under various noise levels, network scales, and complex propagation environments, with localization results approaching the theoretical limits. Compared with classical localization methods such as relaxation-based and weighted least squares methods, the DIJE method shows significant advantages in both localization accuracy and robustness. It effectively meets the engineering requirements for real-time indoor positioning. In summary, the DIJE method effectively addresses the dual-parameter uncertainty problem in RSSI-based indoor localization, providing an efficient and robust technical solution for achieving high-accuracy, low-latency RSSI localization in complex indoor environments.

Although the proposed DIJE method demonstrates satisfactory performance both theoretically and experimentally, certain limitations remain that warrant further optimization and extension. On the one hand, the approximation introduced in the derivation of the DIJE method inevitably leads to performance degradation under high-noise conditions, indicating that there is still room for improvement in localization accuracy in high-noise scenarios. On the other hand, the current method assumes that different APs share a common path-loss exponent. This assumption may limit its applicability in complex real-world indoor environments where propagation characteristics exhibit significant heterogeneity. To address the aforementioned limitations, future research will focus on two key directions. First, more accurate model approximation methods or compensation mechanisms will be explored to mitigate the performance loss caused by approximation errors under high-noise conditions. Second, the method will be extended and optimized to accommodate practical scenarios with heterogeneous path-loss exponents, thereby comprehensively enhancing the generality and engineering application value of the DIJE method.

## Figures and Tables

**Table 1:** List of key notations.

Notation	Explanation	Notation	Explanation
*N*	Number of the APs	$r_{n}$	RSSI of the *n*th AP
$r_{0}$	RSSI at reference distance $d_{0}$	η	Path-loss exponent
$d_{n}$	Distance between the *n*th AP and the target	u	Location of the target
$s_{n}$	Location of the *n*th AP	$\sigma^{2}$	Variance of the shadow fading term

**Table 2:** For each algorithm, the 95% confidence interval and average runtime.

Algorithm	95% Confidence Interval Half-Width/m^2^	Average Runtime/ms
Proposed	0.24	2.44
RSDPE	0.76	842.73
MLE-TLLS	0.22	30.01
SOCP3	2.35	4352
LCJE	0.35	2.24

**Table 3:** TMSE_p_ of different localization algorithms on the public dataset.

Algorithm	Proposed			ANLS		RSDPE	MLE-TLLS
$\eta_{0}$	1.2	1.9	2.5	1.9	1.5	1.5	1.5
TMSE_p_/m^2^	1.7	1.4	1.5	1.9	2.7	8.7	9.9

**Table 4:** TMSE_p_ of different localization algorithms on the real-world complex indoor environment dataset.

Algorithm	Proposed	MLE-TLLS	SOCP3	LCJE	RSDPE	ANLS
TMSE_p_/m^2^	16.7	17.1	19.0	18.6	20.2	23.5

## Data Availability

The public datasets used in this study are available in the cited references [23,25].

## Abbreviations

The following abbreviations are used in this manuscript:

IoT	Internet of Things
DFL	Device-free localization
CSI	Channel state information
RSSI	Received signal strength indication
AP	Access point
MLE	Maximum likelihood estimation
CRLB	Cramér–Rao Lower Bound
SOCP	Second-order cone programming
SDP	Semidefinite programming
DIJE	Differential Iterative Joint Estimation
NLOS	Non-line-of-sight
i.i.d.	independent and identically distributed
WSN	Wireless sensor network
GLS	Generalized least squares
TMSE	Total mean square error
FIM	Fisher Information Matrix
TCRLB	Total Cramér–Rao Lower Bound
SNR	Signal-to-noise ratio
